# A New Capacitance Sensor for Measuring the Void Fraction of Two-Phase Flow Through Tube Bundles

**DOI:** 10.3390/s20072088

**Published:** 2020-04-08

**Authors:** Wael Ahmed, Adib Fatayerji, Ahmed Elsaftawy, Marwan Hassan, David Weaver, Jovica Riznic

**Affiliations:** 1 School of Engineering, University of Guelph, Guelph, ON N1G2W1, Canada; afatayer@uoguelph.ca (A.F.); elsaftaa@uoguelph.ca (A.E.); mahassan@uoguelph.ca (M.H.); 2 Faculty of Engineering, McMaster University, Hamilton, ON L8S4L7, Canada; weaverds@mcmaster.ca; 3 Canadian Nuclear Safety Commission, Ottawa, ON K1P5S9, Canada; jovica.riznic@canada.ca

**Keywords:** two-phase flow, void fraction measurement, capacitance sensor, void fraction, flow patterns, tube bundle

## Abstract

Evaluating the two-phase flow parameters across tube bundles is crucial to the analysis of vibration excitation mechanisms. These parameters include the temporal and local variation of void fraction and phase redistribution. Understanding these two-phase parameters is essential to evaluating the stability threshold of tube bundle configurations. In this work, capacitance sensor probes were designed using finite element analysis to ensure high sensor sensitivity and optimum response. A simulation-based approach was used to calibrate and increase the accuracy of the void fraction measurement. The simulation results were used to scale the normalized capacitance and minimize the sensor uncertainty to ±5%. The sensor and required conditioning circuits were fabricated and tested for measuring the instantaneous void fraction in a horizontal triangular tube bundle array under both static and dynamic two-phase flow conditions. The static calibration of the sensor was able to reduce the uncertainty to ±3% while the sensor conditioning circuit was able to capture instantaneous void fraction signals with frequencies up to 2.5 kHz.

## 1. Introduction

Two-phase flow through a complex geometry is found in many shell-and-tube heat exchangers including nuclear steam generators, reboilers, evaporators and condensers. High-velocity flow across the tubes of such components is known to produce excessive vibrations due to a variety of flow-induced vibration mechanisms, the most serious of which is fluidelastic instability (FEI) [1]. In extreme cases, FEI can produce massive tube failures after only a few hours of service, while in other cases, the failures may be long-term due to fretting wear and fatigue [2]. Evaluating the two-phase flow parameters across tube bundles is crucial to the analysis of vibration excitation mechanisms. These parameters include the temporal and local variation of void fraction and phase redistribution and considered to be essential to determine damping ratio, hydrodynamic mass and fluidelastic instability constants in order to evaluate the stability threshold of tube bundle configurations. In addition, a reliable prediction of the two-phase flow parameters is essential for the development of more efficient, safe and compact steam generating equipment [3].

The void fraction, or volume fraction, is one of the critical parameters that characterizes the nature of the two-phase flow. It is defined as the fraction of volume that is occupied by the gas phase. It has been challenging for researchers to develop experimental techniques in order to measure such a physical quantity. The prediction of the void fraction in a system in which a two-phase flow is present is the most important step since it determines the performance of the system. Two-phase flows have been known to be quite complex, and in most cases, exist in various patterns that are chaotic and irregular. This contributes greatly to the various types of uncertainties in the experimental techniques that have been developed. Although many correlations have been developed for the void fraction, the accuracy of these correlations highly depends on the reliability of the data, the range over which the data is obtained, and the measurement techniques used. In recent decades, numerous techniques have been developed for measuring the void fraction in order to ascertain precise and reliable data for both local and averaged void fractions [2].

Despite the importance and the prevalence of two-phase flow in tube bundles, the number of publications and the amount of research available to consider all physical two-phase flow parameters in tube bundles is considerably lower than those for pipe flow. This lack of publications is partly due to the difficulty of performing experiments and obtaining reliable void fraction measurements in such complex geometry. For example, Taylor and Pettigrew [4] studied two-phase flow-induced vibration (FIV) to determine the critical velocity for fluidelastic instability in tube bundles. This paper emphasized how the analysis of flow-induced vibrations is strongly dependent on the void fraction and the flow pattern but it did not provide a detailed and reliable evaluation of two-phase void fraction measurements.

Noghrehkar et al. [1] performed experiments to identify flow patterns across triangular and square tube bundles. The flow patterns were identified visually through the side window and also by using void fraction measurements collected from a resistive probe installed in the core of the bundle. Under certain conditions, Noghrehkar et al. [1] observed a bubbly flow pattern near the window while the resistive signal approach identified intermittent flow away from the wall. These findings might indicate that predictive methods based on visual observations may not give an accurate flow pattern identification, especially away from the wall. On the other hand, Kanizawa [5] objectively identified the flow pattern inside a normal triangular tube bundle using signals from pressure transducers and capacitive sensors. The *k*-means clustering method, which was based on signals from the pressure transducers and the capacitive sensors, was used to identify the flow pattern. Results were compared with predictive methods available in the literature and showed a satisfactory agreement for identifying flow patterns.

Xu et al. [6] and Schrage et al. [7] used the quick closing valve technique for measuring the volumetric average void fraction in both an external and internal two-phase flow. In this technique, two quick closing valves at both ends of the flow channel are simultaneously closed trapping the fluid in the volume. The volumetric void fraction is then determined by measuring how much of the liquid is occupying the boundary. The quick closing valves method does not, however, give the instantaneous void fraction measurement and cannot be utilized when FIV data is needed. To implement this technique, one must also stop the flow, which is not always convenient.

Flow visualization and image processing techniques have been employed for void fraction measurements. High-speed imaging and good lighting are essential for this technique to work. This technique can be somewhat subjective as different contrast thresholds can be used to distinguish between the two phases. In addition, an image captured at a particular depth or focal point is influenced or cluttered by the flow at a different focal point. Iwaki et al. [8] conducted a study in which they estimated the void fraction by analyzing flow images across square and triangular tube bundles. From this analysis, Iwaki estimated the size and concentration of the bubbles.

Ursenbacher et al. [9] measured the void fraction of a refrigerant flowing through a horizontal tube. This was done by mixing a dye with the refrigerant and then illuminating it using a laser sheet. The laser sheet was placed perpendicular to the tube axis. High-speed images were captured and analyzed to extract the measurements. It was found that the refraction of light at the gas–liquid interface may introduce some noise to the measurement.

Gamma-ray densitometry, developed in the 1950s, is a non-intrusive measurement method that is commonly used for a line-averaged void fraction and is considered one of the most accurate void fraction measuring techniques. In this technique, gamma rays are directed from one side of the cross-section and received in a detector placed on the other side. The difference in the intensity between the emitted and the received rays are related and calibrated to the average void fraction along the ray line. Feenstra et al. [10] used gamma-ray densitometry to measure the void fraction across tube bundles. This method works because the absorption of the gamma rays is dependent on the density and the void fraction of the fluid. Despite its accuracy, this technique has many disadvantages including high costs, safety concerns and a slow response time.

Capacitance sensors can be designed as either intrusive or non-intrusive instruments to measure the volume average void fraction. Simplicity and cost are the main advantages of the capacitance sensor. This approach also gives a real-time measurement of the void fraction. Unlike the resistive probe sensor, the capacitance sensor works well with non-conductive water. The capacitance values of the sensor are highly dependent on the flow pattern [11]; for example, a bubbly flow with a 10% void fraction can give a higher capacitance value compared to an annular flow with the same void fraction. To get around this issue, the flow pattern has to be identified before determining the void fraction reading. Flow pattern can be predicted by using either flow pattern maps or by interpreting the probability density function (PDF) and the power spectral density (PSD) of the signal [12]. Only two experimental studies were found that used a capacitance sensor to measure the void fraction in tube bundles [13,14].

Kanizawa [13] performed void fraction measurements for a bubbly flow inside a vertical tube bundle using a capacitance sensor. The capacitance sensor was made from a pair of electrodes installed facing each other. Each electrode was surrounded by two other shielding electrodes to minimize noise. The probes were molded in epoxy to insulate them from the water and to prevent the flow of electrons; hence, a stable electric field could be produced. Kanizawa [13] used the normalized capacitance signal under quiescent liquid and low superficial gas velocities with void fraction measurements found using the gravimetric method. The gravimetric method only works for small bubbly flows, which occur under very low superficial air and liquid velocities. Kanizawa’s [13] results show that for small bubbles, the normalized capacitance was slightly smaller than the void fraction measurement using the gravimetric method. The capacitance signal was linear for a small bubbly flow. Similarly, Watanabe [14] developed a capacitance void fraction measurement method for a Boiling Water Reactor (BWR) test and compared the data with void fraction measurements using the quick closing valve method to validate the measurements. The capacitance probes were placed outside the casing of the tube bundles. An LRC (inductor, resistor and a capacitance) circuit was used to measure the frequency response of the capacitor. Based on the frequency response, the capacitance value was computed. Results from these experiments showed a very good agreement between the void fractions measured using the capacitance sensor and the quick closing valve method. The void fraction values from the capacitance sensor were 4% to 8% larger than the quick shut method. The difference between the two methods decreased with an increasing mass flow ratio or void fraction. Chen et al. [15] performed finite element method (FEM) analysis to compute the effective permittivity of a two-phase disordered composite material as a function of the volume fraction. The simulation was done for three different dielectric contrasts with the highest contrast being one to thirty. The inclusion material was arranged randomly in a 10 × 10 × 10 cube cell. Ten random distributions of the inclusion material were generated for each void fraction. Results show that the highest permittivity contrast ratio had the highest standard deviation. The highest maximum relative error to the mean was about 5%.

The main objective of the current study is to validate the operating principles of a newly designed sensor for measuring the void fraction in a tube bundle. This sensor is designed to provide detailed information on two-phase flow distribution within tube bundle arrangements with the capability of measuring both the local and average void fraction. Finite element analysis was used to optimize the sensor design and maximize its response. Simulation results were also used to calibrate the sensor for an air–water mixture by scaling the normalized capacitance value to match the actual void fraction. Moreover, the operation of the sensor was expanded to other applications and fluids by testing it for applications involving the refrigerant R123 commonly used for two-phase flow lab experiments.

## 2. Working Principles and Sensor Design

A capacitor sensor was designed, modeled and built to measure the volume-averaged void fraction of a vertically upward flow through a thirty-degree triangular tube bundle. The design of the capacitance sensor and the sensor configurations are discussed in detail in this section. Moreover, a two-dimensional computational finite element model is presented along with the computational mesh. These computational results were used to study the response of the sensor and relate the normalized capacitance signal to the void fraction. A capacitor is a device used to store an electrical charge, measured in farads, which is the change in the electric charge (*q*) per unit change in the electric potential (*V*):

(1) $$C=\frac{q}{V}$$

Capacitors are made of an anode and a cathode plate (combined as electrodes) separated by a dielectric material. The dielectric material is an insulating material that can be polarized by applying an electric field. This electric field is generated between the anode and cathode terminals because of the potential electric difference between them. The electric field polarizes the dipoles in the dielectric material by rotating the charged molecules making the positive side in line with the electric field. The energy is stored in the dielectric material through this polarization process. The electric field can be thought of as a force while the charged molecules are a spring. Polarizing the molecules is like compressing the spring and storing the potential energy. Just like different materials have different stiffnesses, dielectrics have different permittivities. The permittivity of a dielectric is a measure of the resistivity encountered by the electric field in a medium, with units of farads per meter or Newtons per meter squared. Dielectric materials are characterized by their relative permittivity, which is also known as the dielectric constant. The dielectric constant is the permittivity of a material relative to the absolute permittivity of the free space, which is 8.85 × 10^−12^ F/m. Permittivity is not always constant as it is affected by many variables, such as the position in the medium, the frequency of the field applied, the humidity and the temperature.

**Figure 1:**
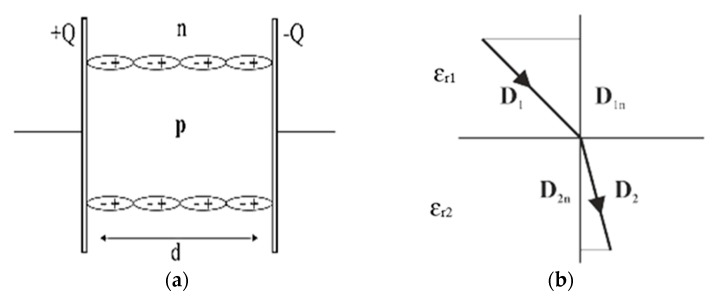
(**a**) Polarized atoms inside an electric field; (**b**) electric displacement field at the interface of different materials.

Dielectrics can be either polar or non-polar. Water is an example of a polar dielectric. In a water molecule, the hydrogen atoms have a partial positive charge while the oxygen atom has a partially negative charge. Initially, the water dipoles are randomly aligned. When the water molecules are inserted inside an electric field, the dipoles align themselves with the direction of the electric field. Non-polar dielectrics are composed of atoms with no permanent dipole moments. The atoms are made of positively charged nuclei with negatively charged electrons orbiting around them. The electrons are not static but are instead continuously orbiting the nuclei in a cloud. The negative charge of the electrons is equal to the positive charge of the nuclei. This results in neutral atoms with no net charge. In the absence of an applied electric field, the center of the negatively charged cloud of electrons coincides with the center of the positively charged nuclei. When an electric field is applied, the center of the positive charge gets pushed in the direction of the electric field while the center of the negative charges gets pulled in the opposite direction. This results in a dipole moment like the one in Figure 1a. The dipole moment of a non-polar dielectric can be represented by a pair of equal and opposite charges separated by a distance.

Since an air bubble passing between two electrodes is a non-polar dielectric, the individual atoms are polarized and the centers of the charges are stretched away from each other. On a macroscopic scale inside the air bubble, the electric effects of the dipoles cancel out because the net charge inside the bubble is zero. The positive dipole of an atom in the air faces the negative dipole of another atom resulting in a zero-charge density inside the bubble. The surface of the bubble, however, is positively charged on one side and negatively charged on the other. This is because there are no atoms on the poles to neutralize the charges. The positive surface of the bubble is partially in line with the electric field as shown in Figure 1a. The polarized molecules create a small electric field opposite to the applied electric field, which reduces the overall electric field in the capacitor. This also reduces the potential across the terminals. When a constant voltage source is applied to the terminals, a charge begins to accumulate on the surface of the terminals to increase the potential, thus increasing the electric field in the dielectric.

Now, consider two dielectric materials that are connected by a surface interface between them as shown in Figure 1b. The upper and the lower normal surface vectors are *A*_1_ and *A*_2_, respectively:

(2) $$A_{1}=-A_{2}$$

As both dielectrics do not have free charges, the electric displacement (*D*) flux is zero. As the area is perpendicular to the field, so the integral over this section is zero. In addition, the integral on the face that is outside the capacitor where *D* is zero.

(3) $$\oint DdA=D_{1}A_{1}+D_{2}A_{2}=0$$

(4) $$D_{1}A_{1}=-D_{2}A_{2}$$

The electric displacement can be obtained using the dot product between the electric displacement vector and the normal surface vector as:

(5) $$D_{1n}A_{1}=D_{2n}A_{2}$$

In order to determine the electric field at the contact region, it is assumed that a narrow loop encloses the interface of the dielectrics. The part of the loop parallel to the interface is denoted by s and −s vectors. The part of the loop normal to the surface is insignificant compared to the parallel component. Now, the closed loop integral of the electric field (*E*) is zero:

(6) $$\oint Edr=sE_{1}-sE_{2}=0$$

(7) $$sE_{1}=sE_{2}$$

Then, the electric field tangent to the interface is determined using the dot product between the vector *s* and the electric field.

(8) $$sE_{1t}=sE_{2t}$$

### Sensor Design

**Figure 2:**
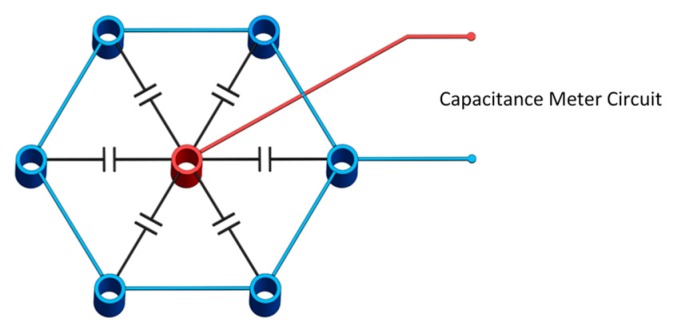
Single-sensor configuration.

**Figure 3:**
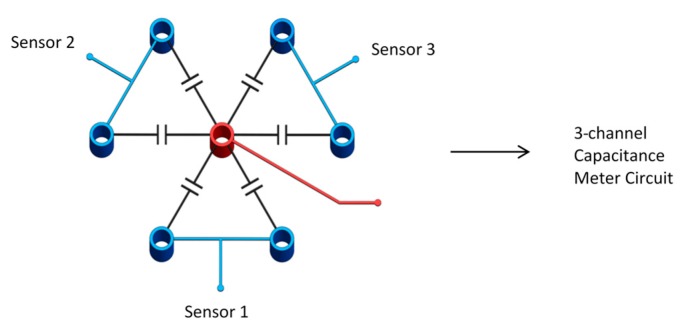
Three-sensor configuration.

The present capacitance sensor consists of seven cylindrical copper electrodes arranged in a bundle as shown in Figure 2 and Figure 3. Each electrode is made of a 12.7 mm diameter copper tube filled with resin. Copper rings are placed on the top and the bottom of each electrode to shield them from any surrounding noise. The surface of each electrode is coated with a thin layer of varnish to insulate it from the liquid. This allows the electric field to pass while blocking the electric current.

As the gas–liquid mixture flows through the electric field between the electrodes, the total dielectric constant changes depending on the gas-to-liquid ratio. The principle behind this is the considerable difference in the dielectric constant between the gas and liquid, which are 1 and 80, respectively. As the air-to-water ratio changes, the capacitance (C) also changes according to the relation:

(9) $$C\propto \left(\varepsilon_{0}*\varepsilon_{r}\right)$$

where $\varepsilon_{0}$ is the primitivity of the free space and $\varepsilon_{r}$ is the relative primitivity.

The sensor calibration is performed by taking two capacitance measurements: one at a 0% void fraction and another at a 100% void fraction. Those two points are then used to calculate the total percentage of the void fraction from the dynamic capacitance signal.

Two different electrode configurations were used: a single-sensor configuration and a three-sensor configuration. In the single-sensor configuration shown in Figure 2, the center electrode is connected to one terminal of the meter circuit while the other six outer electrodes are connected to the second terminal; therefore, this arrangement has six capacitances between the outer electrodes and the center one.

In the three-sensor configuration shown in Figure 3, each sensor consists of two outer adjacent electrodes connected together to form two capacitances with the center electrode. The meter circuit used for this configuration has three input channels to measure the three sensors.

## 3. Experimental Setup for Sensor Evaluation

**Figure 4:**
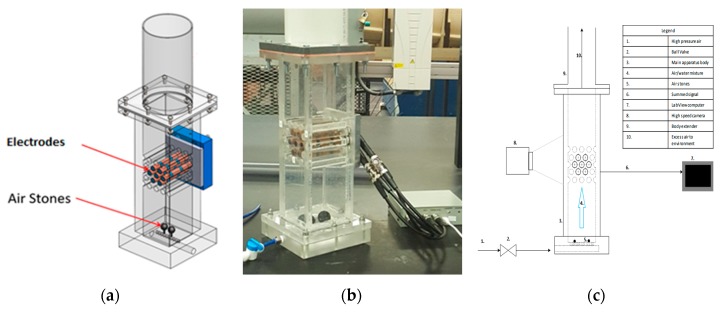
(**a**) Three-dimensional (3D) model of the apparatus; (**b**) actual picture of apparatus used for the experiment; (**c**) flow diagram.

Two experimental setups were built to validate the operation of the capacitance sensor. The first setup was built for a proof of concept of the sensor under air–water operating conditions, while the other setup was designed to be used to investigate refrigerant R123 used for two-phase flow-induced vibrations research. The tube bundle used for the first setup was placed inside a rectangular duct made of acrylic. The bundle had five rows of tubes and four columns. Half of the tubes were placed on the wall of the duct. The tubes in the bundle had a diameter of 12.7 mm and a 1.5 pitch to diameter ratio. The capacitance sensor was placed in the center of the tube bundle in a honeycomb pattern. Figure 4a,b shows a 3D model and the actual setup used in the experiment.

An air–water mixture was the medium used, and, therefore, the electrodes were coated in a thin layer of varnish to insulate them from the conductive tap water. The apparatus was filled with water until all the electrodes were fully submerged, and the apparatus was almost full. Air was pumped in from the bottom via an air-line at various flow rates. Air was passed through the holes and the air stones at the bottom to create a uniform bubbling flow past the electrodes. Flow rates of 10, 20, 30 and 40 liters per minute (L/min) were used to gather data. A flow diagram of the system is shown in Figure 4c.

**Figure 5:**
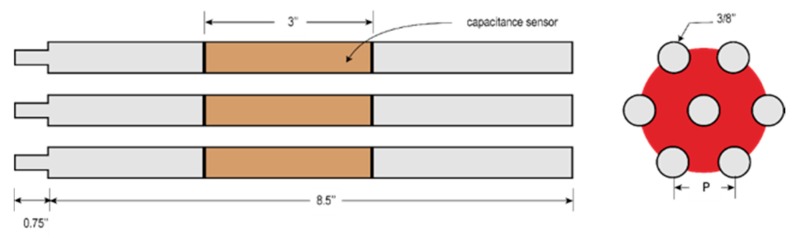
The sensor geometrical parameters.

In the second setup, castor oil and air were used to simulate the dielectric performance of the two-phase gas–liquid of refrigerant flow, castor oil and air were found to have the same dielectric constants as the liquid and gas phases of the refrigerant R123. This setup is shown in Figure 5. The bundle had a pitch P = 0.56 inch and a diameter of 3/8 inch. The air flow rate was measured using a mass flow meter before it entered the test section, while the stagnant castor oil was kept inside the test section. The oil level was kept high such that it covered all of the tube before the air was injected. All electrodes were connected to the acquisition system to capture the response of the sensor.

### 3.1. Conditioning-Metering Circuit Design

**Figure 6:**
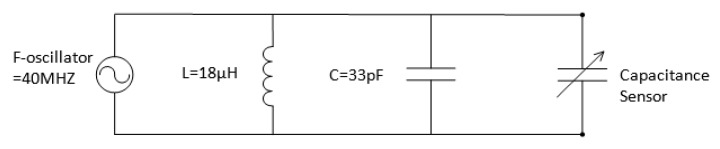
LC oscillator circuit connected parallel to the capacitance sensor.

The meter circuit used the LC approach to measure the capacitance at a maximum sampling rate of 2500 samples per second and a resolution of 28 bits. This sampling frequency is considered to be at least three to four times the expected frequency of the two-phase flow exists in the present application. The capacitance was measured using a frequency-to-digital converter module FDC2214 EVM made by Texas Instruments. The module had a built-in LC resonator circuit, which consisted of a parallel LC circuit and an oscillator. The capacitance sensor was connected parallel to the LC circuit as shown in Figure 6.

As the capacitance value of the sensor changed, the total capacitance of the LC circuit also changed causing the resonance frequency (f) to change. The relationship between the resonance frequency and capacitance (C) and inductance (L) is given by:

(10) $$f=\frac{1}{2\pi \sqrt{L*\left(C+C_{sensor}\right)}}.$$

**Figure 7:**
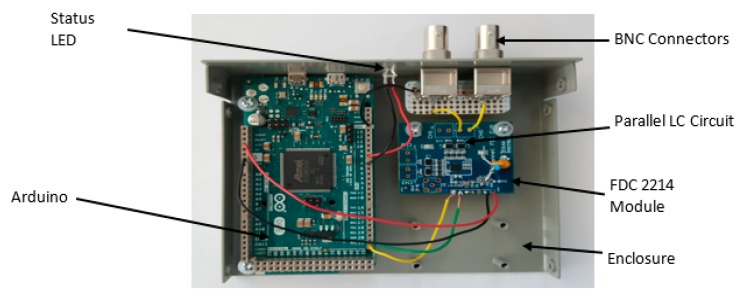
Actual image of the single-channel meter circuit.

The FDC measured this change as resonance frequency and output a digital value that was proportional to this frequency. This value was then converted to an equivalent capacitance value by the Arduino. The Arduino acted as an interface between the FDC2214 and a LabVIEW virtual instrument running on a host computer. The Arduino was responsible for the serial communication, configuring the FDC2214, reading the values supplied by the FDC2214 and calculating the capacitance. An actual image of the complete single-channel meter circuit is shown in Figure 7.

### 3.2. Optimizing the Sensor Geometry and Response

A 2D finite element model was implemented to optimize the geometrical parameters of the sensor to maximize the capacitance response of the sensor for the entire range needed for void fraction. The simulations were carried out using COMSOL 5.1 that is commonly used for multiphysics simulations. The static simulations were done using the electrostatic physics module. Using Equations (13)–(15), the normalized capacitance was defined in order to obtain a linear relationship between the overall capacitance and the void fraction (α) as explained in details by Elsaftawy et al. [16]:

(11) $$C=\alpha C_{gas}+\left(1-\alpha \right)C_{liquid}$$

(12) $$\alpha =\frac{C-C_{liquid}}{C_{gas}-C_{liquid}}$$

(13) $$C_{norm}=\frac{C-C_{liquid}}{C_{gas}-C_{liquid}}$$

**Figure 8:**
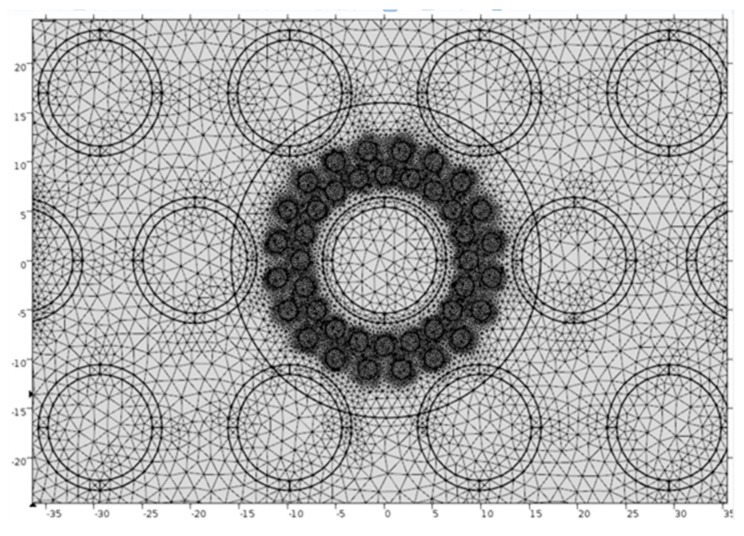
Computational mesh for solving the electric field.

The normalized capacitance value was used to measure the accuracy and the deviation in the measured void fraction from that of the actual void fraction. A 2D model was built to resemble the tube bundles and the sensor in the bundle. The acrylic material was assigned to the non-sensor tubes in the domain. The sensor electrodes were modeled using a circle with three layers. The inner layer was made of resin. The middle copper layer had a thickness of 1 mm while the outer insulating layer had a thickness of 0.1 mm. A blank material with a relative permittivity equal to that of the varnish was applied to the outer layer of the electrode. Air bubbles were modeled as either perfect circles or ellipses. For the simulations, the bubbles were distributed using either a rectangular or a circular array. Water covering the rest of the domain was assumed to have an isotropic and constant relative permittivity of 80.1, which is the permittivity of water at 25 °C. The outer sensor electrodes were set at 5V while the center electrode was grounded. A zero-charge boundary condition was applied to the domain boundary. An adaptive meshing technique, shown in Figure 8, was used for this simulation. The domain was discretized using 200,000 triangular mesh elements. The elements were mostly concentrated in the sensor region especially around the boundary of each bubble. Ninety percent of the elements were less than 3 × 10^–4^ mm^2^. Doubling the number of elements to 400,000 made no changes to the simulation results.

The basis of all electromagnetic analysis revolves around solving Maxwell’s equations subject to satisfied boundary conditions. Maxwell’s equations relate the fundamental electromagnetic quantities using differential or integral equations. The fundamental quantities of interest for computing the capacitance are the electric field intensity, ***E***, the electric displacement or electric flux density, ***D***, and the electric charge density, (*ρ*) [17]. The finite element method (FEM) approach used in solving the electrostatic problem uses the differential forms of Maxwell’s equations. The electric field is computed using Equation (14). The electric field is found by taking the negative of the divergence of the electric potential. The electric displacement is then calculated using Equation (15). Lastly, the charge density is then found using Equation (16).

(14) E=−∇V

(15) $$D=\varepsilon_{0}\varepsilon_{r}E$$

(16) ∇·D=ρ

The global capacitance value of the domain is computed using the energy approach. The energy density, *We*, is calculated using Equations (17) and (18):

(17) $$\delta We=\frac{1}{2}\iint \varepsilon \left[{\left(\frac{\partial V}{\partial x}\right)}^{2}+{\left(\frac{\partial V}{\partial y}\right)}^{2}\right]dxdy$$

(18) We=∑δWe

The global capacitance can then be found using the total electric energy in the domain and the difference in the electrical potential between the two terminals. The global capacitance is evaluated using Equation (19):

(19) $$C=\frac{2We}{{\left(V_{1}-V_{o}\right)}^{2}}$$

Boundary conditions are necessary to solve Maxwell’s equations. At the interface between water and air, the following boundary conditions are applied:

(20) $$n_{2}\times \left(E_{1}-E_{2}\right)=0$$

where *n*_2_ is the outward normal from air and $\rho_{s}$ is the surface charge density. As was mentioned earlier, the electric field and the electric displacement are zero for a perfect conductor. Using this fact, the following boundary conditions for the interface between the electrical rods and the dielectrics are applied:

(21) $$-n_{2}\times E_{2}=0$$

(22) $$-n_{2}\cdot D_{2}=\rho_{s}$$

## 4. Results and Discussion

### 4.1. Computational Model Validation

**Figure 9:**
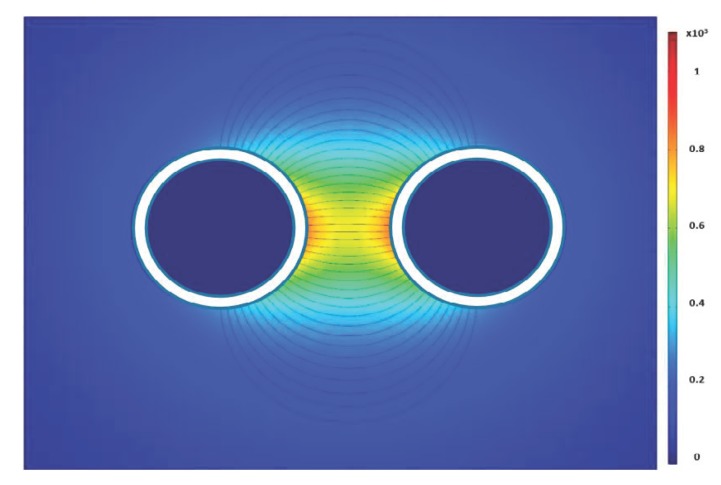
Capacitance contour plot of two parallel cylindrical capacitors.

The computational model was validated using a simple capacitor design with two parallel cylinders, as shown in Figure 9. The capacitance of two parallel cylinders with opposite applied potential was simulated and compared with the empirical correlation, seen in Equation (23) [17]:

(23) $$C=\frac{\pi \varepsilon l}{\ln\left(\frac{d}{2a}+\sqrt{\frac{d^{2}}{4a^{2}}-1}\right)}$$

where *l* is the cylinder length, *d* is the distance between the centers of the two cylinders and *a* is the radius of each cylinder. The capacitance values of the simulated results are compared with experimental data for all liquid and all gas values. The comparison between experiments and finite element simulation shows an RMS error less than ±1%, which is considered reasonable for optimizing the sensor design using the finite element simulation tool.

### 4.2. Optimizing the Sensor Design

**Figure 10:**
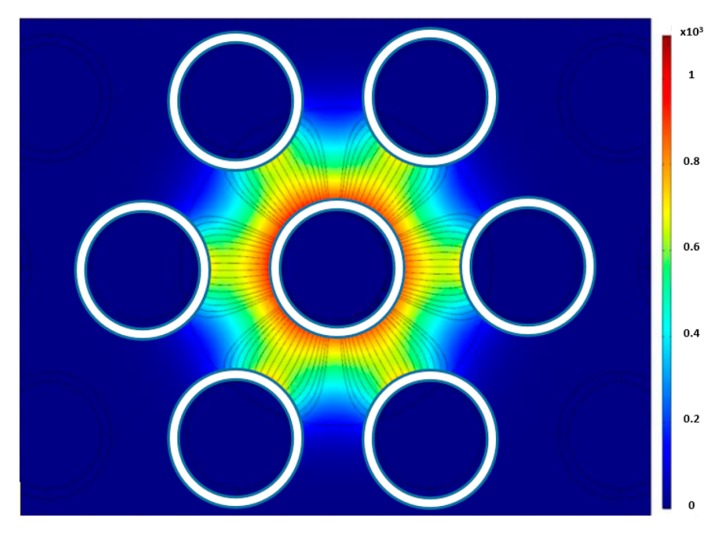
Electric field contour for the case with no air bubbles.

Numerous simulation tests and case studies were performed to determine the sensitivity of the sensor to the void fraction and the bubble distribution and sizes. Figure 10 shows the electric field strengths and the electric field lines when there are no bubbles in the domain. The electric field contour shows that the electric field is strongest in the center. This is because all the electric field lines from the surrounding electrodes converge to the negative center electrode. The electric field inside the enclosed circle ranges from 180 V/m to 998 V/m. The average electric field in the enclosed circle is 542 V/m with a standard deviation of 232 V/m. The volumetric resolution of the sensor is the area enclosed by the circle outlined in black. Any bubbles outside this domain will have no effect on the sensor response.

**Figure 11:**
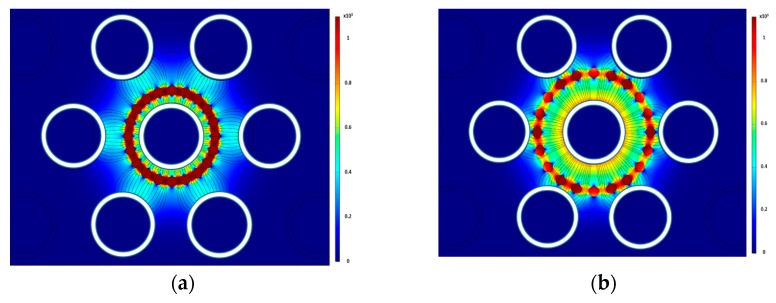
Electric field contour for bubble diameters of (**a**) 7 mm and (**b**) 9 mm.

**Figure 12:**
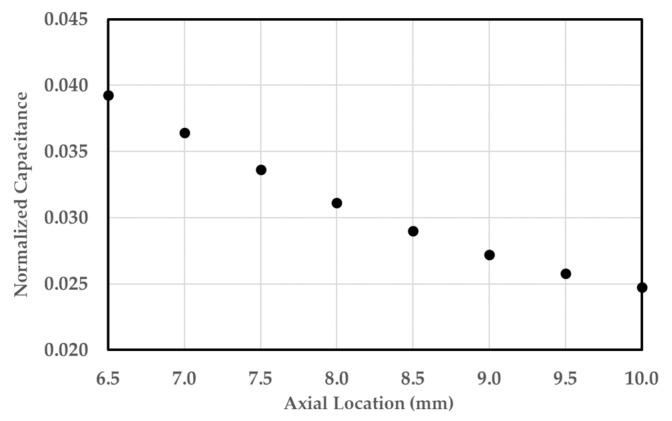
Effect of the axial location of the bubbles on the normalized capacitance.

To test the effects of axial bubble location, a simulation study was done using a cluster of bubbles at various distances from the center. Figure 11 shows the electric field strength when the bubbles are 7 mm and 9 mm from the center. The sensor is more sensitive to bubbles located near the sensor compared to bubbles further away. This is because the electric field is strongest in the center since all of the electric field lines coming from the outer electrodes converge into the central electrode. Figure 12 shows the linear response of the sensor interims of normalized capacitance calculated by Equation (24) of the bubble cluster as a function of the distance far from the center.

(24) $$C_{norm}=\frac{C-C_{liquid}}{C_{gas}-C_{liquid}}$$

**Figure 13:**
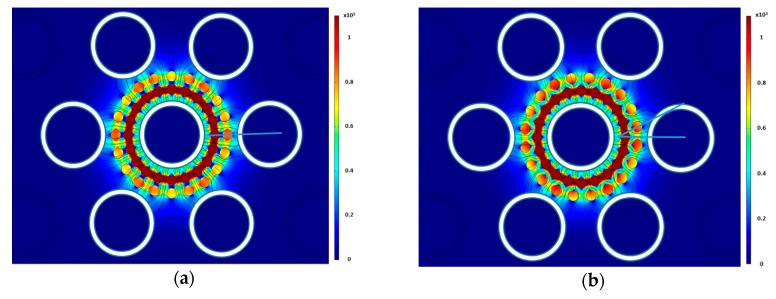
Electric field contour for relative angles between the two sets at (**a**) 0° and (**b**) 10°.

The next set of runs were done to determine the effects of bubble locations relative to other bubbles on the overall normalized capacitance value. Two sets of sixteen air bubbles were created in a circular pattern. The inner set was fixed, and the outer set was varied by rotating the pattern, thus changing the alignment of the bubbles. The two sets of bubbles were perfectly aligned when their relative angle was zero. The normalized capacitance value was recorded for eleven different angles. Figure 13a,b shows the electric field distribution when the relative angle was 0° and 10°, respectively.

**Figure 14:**
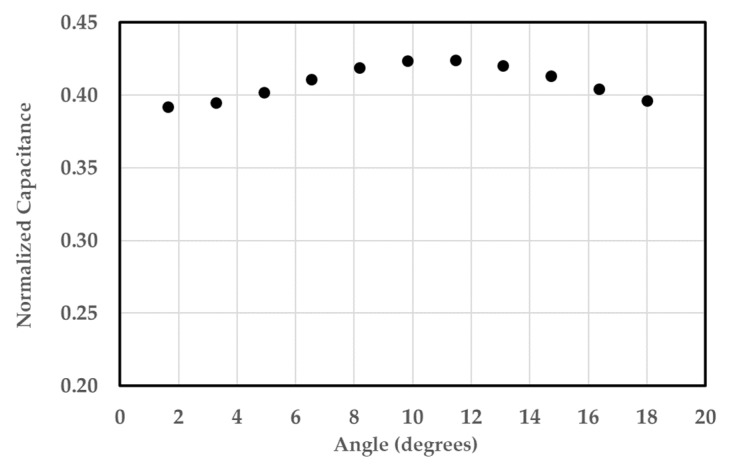
Effect of the relative angle between the two bubble sets on the normalized capacitance.

As shown in Figure 14, the normalized capacitance is lowest when the bubbles are aligned in the same axis and highest when the bubbles are not aligned. The average normalized capacitance value in this study was 0.41 with a standard deviation of 0.01. Around the bubble set, the electric field intensity increased in the regions perpendicular to the electric field lines and decreased in the regions along those lines. The electric field lines going through the water tend to bend around the bubble set. This creates a region with a very low electric field downstream and upstream of each bubble set. The electric field increases on the side of the bubbles. Despite the variations in the capacitance values at different locations, the simulation results show that the electric field in the sensor is nearly axisymmetric. This means that a group of bubbles placed in any of the four quadrants of the sensor will yield the same capacitance value.

### 4.3. Effect of Bubble Distributions on Sensor Response

**Figure 15:**
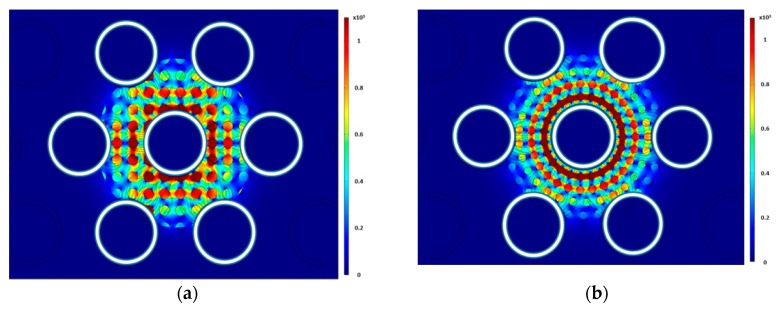
Electric field distribution for (**a**) Arrangement 1 and (**b**) Arrangement 2.

**Figure 16:**
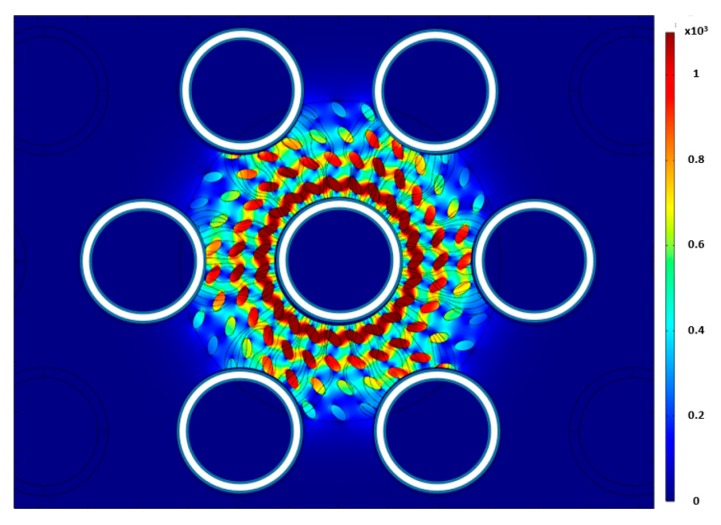
Electric field contour for the distribution of elliptical bubbles.

**Figure 17:**
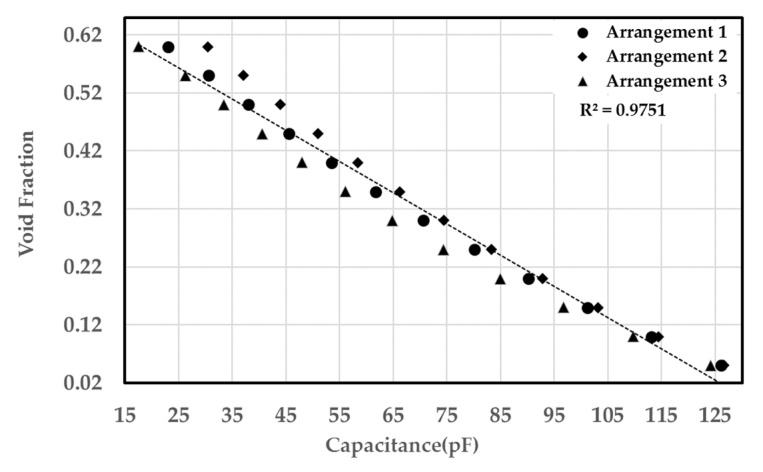
Void fraction versus the normalized capacitance for three uniform bubble distributions.

Three different uniform distributions were simulated to test the sensitivity of the sensor to the bubble distributions and orientation. The first test had circular bubbles that were generated using a rectangular array (Arrangement 1) shown in Figure 15a. In this configuration, each of the other circular patterns was rotated by five degrees to minimize the number of bubbles in each electric field line. The second test had circular bubbles that were generated using a rectangular array (Arrangement 2) shown in Figure 15b. In the third, elliptical bubbles with a 1:2 ratio were used. The bubbles were then rotated by 45 degrees. The elliptical bubbles were distributed using a circular array pattern shown in Figure 16. For each case, the diameter of the bubbles was varied to change the void fraction. Figure 17 shows the void fraction as a function of the normalized capacitance for all three bubble distributions. The correlation coefficient of the present data suggested that the deviation between the capacitance response and the actual void fraction is within ±3%. It was also noted that all three uniform bubble distributions gave the same trend and a similar response. The linear relation between the capacitance value and the void fraction was established using the data points from the simulation. The linear relation has a coefficient of determination of 0.9751. Simulation results, shown in Table 1, show that the sensor is least sensitive under low void fraction conditions; however, the sensor is fairly accurate when the void fraction is more than 15%.

### 4.4. Experimental Validation of the Sensor Response

In this section, the calibrations of the sensor for both air–water and vapor–liquid refrigeration applications are presented for the dynamic measurement of void fraction while monitoring the flow patterns using a high-speed imaging system. The data includes testing of the sensor with its two configurations.

**Figure 18:**
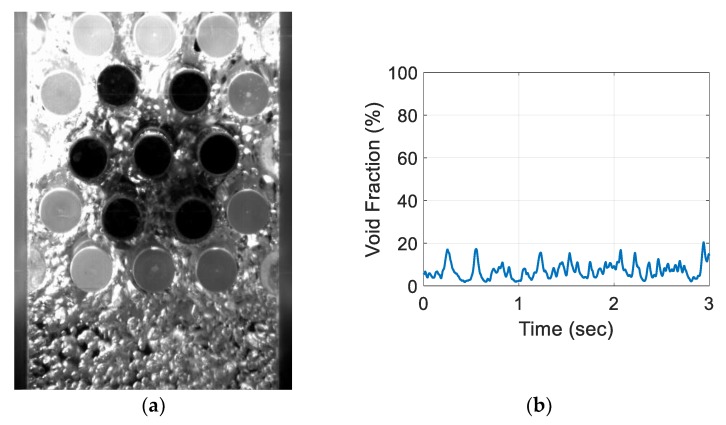
Experimental results for the one sensor configuration at an air flow rate of 5 L/min: (**a**) flow visualization and (**b**) void fraction signal.

**Figure 19:**
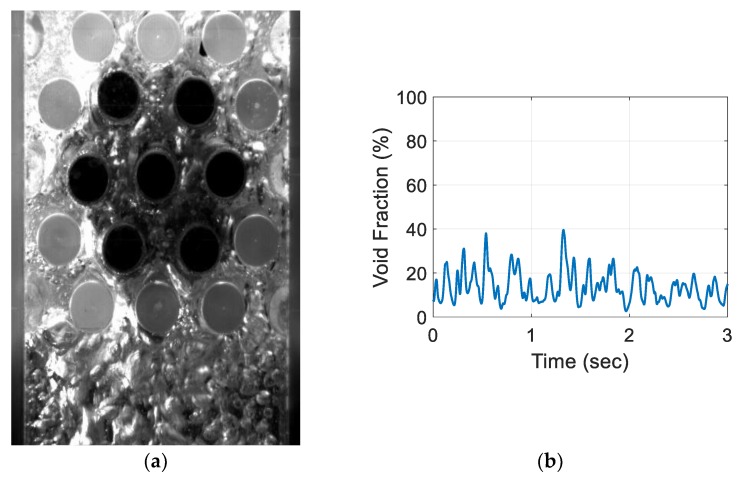
Experimental results for an air flow rate of 10 L/min: (**a**) flow visualization and (**b**) void fraction signal.

**Figure 20:**
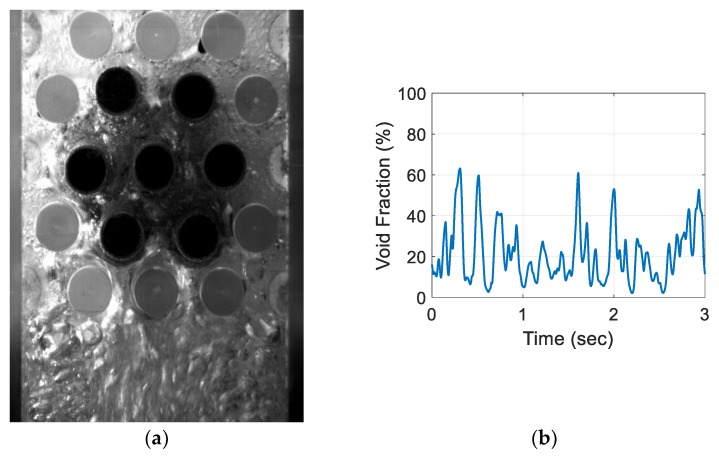
Experimental results for the one sensor configuration at an air flow rate of 20 L/min: (**a**) flow visualization and (**b**) void fraction signal.

**Figure 21:**
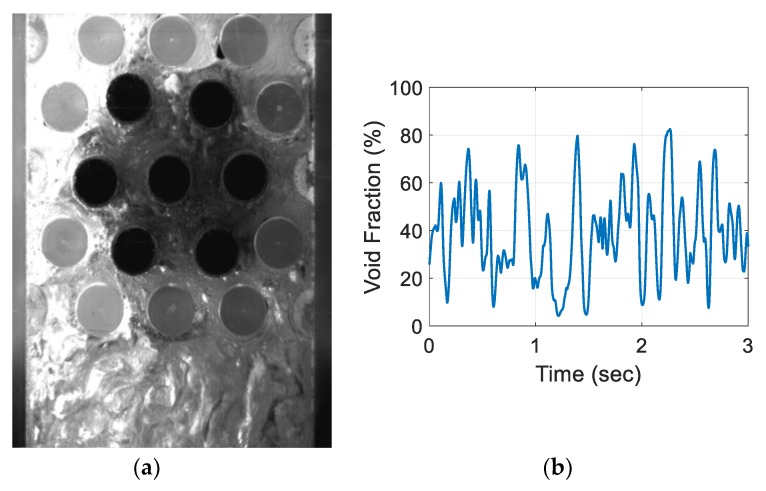
Experimental results for the one sensor configuration for an air flow rate at 40 L/min: (**a**) flow visualization and (**b**) void fraction signal.

**Figure 22:**
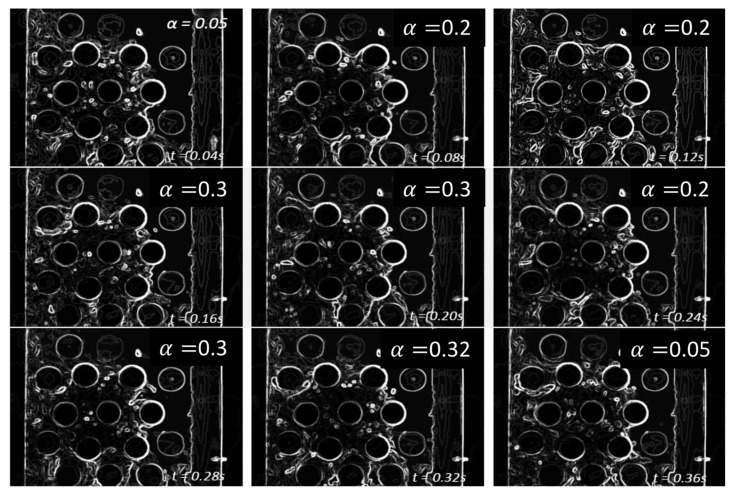
High-speed images of the flow at an air flow rate of 10 L/min and the approximate void fraction found using these images.

For the air–water experiment, the air flow rate was varied while simultaneously recording the sensor signal. The data set for the single-sensor configuration is presented, after which the results from the three-sensor configuration are presented and discussed. As seen in Figure 18, Figure 19, Figure 20 and Figure 21, the fluctuation range in the percentage of the void fraction for the capacitance signal increases for higher air flows. The volume average void fraction is computed by first averaging the normalized capacitance signal and then inputting the value in the calibration curve equation. The calibration curves were derived from the finite element simulation study performed to design the capacitance sensor. This calibration approach assumes that the air was uniformly distributed inside the tube bundle. The data in Figure 19, Figure 20, Figure 21 and Figure 22 show the flow visualization and the void fraction signal for air flow rates of 5, 10, 20 and 40 L/min using the single-sensor configuration. To validate the void fraction measurements, high-speed images of the flow at 10 L/min were captured and the approximate void fraction was estimated using the digital processing of images using MATLAB toolbox. The high-speed images shown in Figure 22 combined with the estimated void fractions validated the measurement approach presented in this paper.

**Figure 23:**
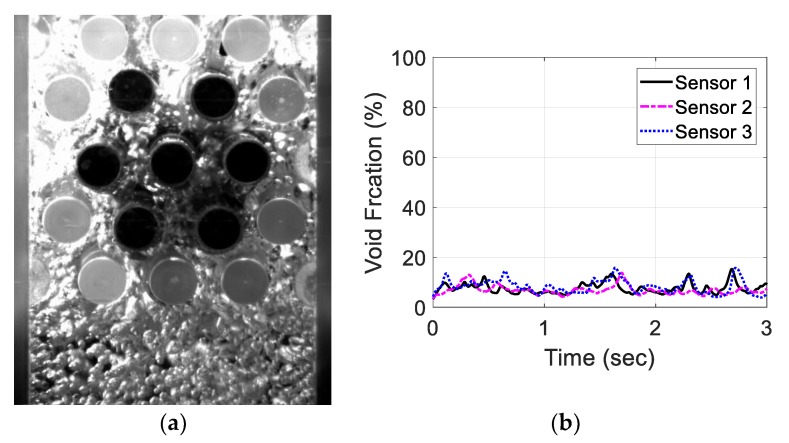
Experimental results for an air flow rate of 5 L/min for the three-sensor configuration: (**a**) flow visualization and (**b**) void fraction signal.

**Figure 24:**
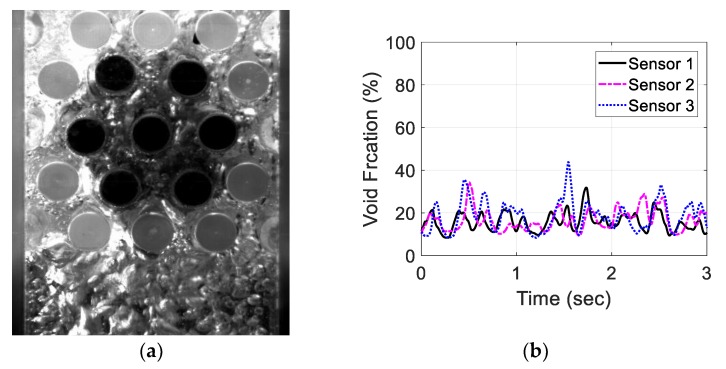
Experimental results for an air flow rate of 10 L/min for the three-sensor configuration: (**a**) flow visualization and (**b**) void fraction signal.

**Figure 25:**
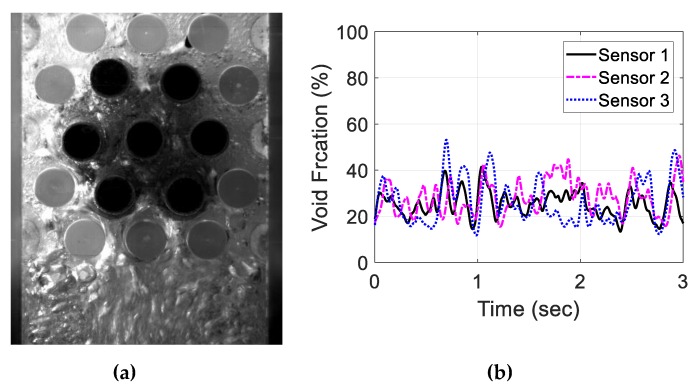
Experimental results for an air flow rate of 20 L/min for the three-sensor configuration: (**a**) flow visualization and (**b**) void fraction signal.

**Figure 26:**
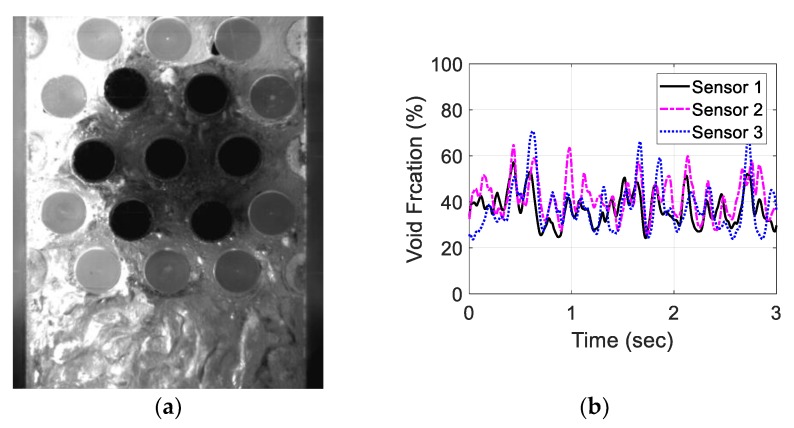
Experimental results for an air flow rate of 40 L/min for the three-sensor configuration: (**a**) flow visualization and (**b**) void fraction signal.

Table 2 shows the average normalized capacitance and the approximate void fraction in the bundle at different air flow rates. The average void fraction is observed to increase as the air flow rate increases:

Moreover, the three-sensor configuration signals were recorded and investigated for the same air flow rates. Figure 23, Figure 24, Figure 25 and Figure 26 show the three void fraction signals for the four air flow rates using the three-sensor electrode configuration. As the air flow rate increased, the fluctuation range in the normalized capacitance signal for the three sensors increased, which is the same trend seen in the single-sensor configuration. The main advantage of the above-mentioned configuration is that it can be used to estimate the distribution of the air bubbles around the tube bundle. The asymmetry of the flow can, therefore, be determined. In the present work, the sampling frequency of each sensor is set to 833 Hz, which is limited by the Arduino maximum frequency. In addition, it is approximately one-third of the sampling rate of the single-sensor configuration, which is 2500 Hz. This sampling frequency is found to exceed the desired flow frequency by 300% which is considered to be adequate for the proposed measurements. 

**Figure 27:**
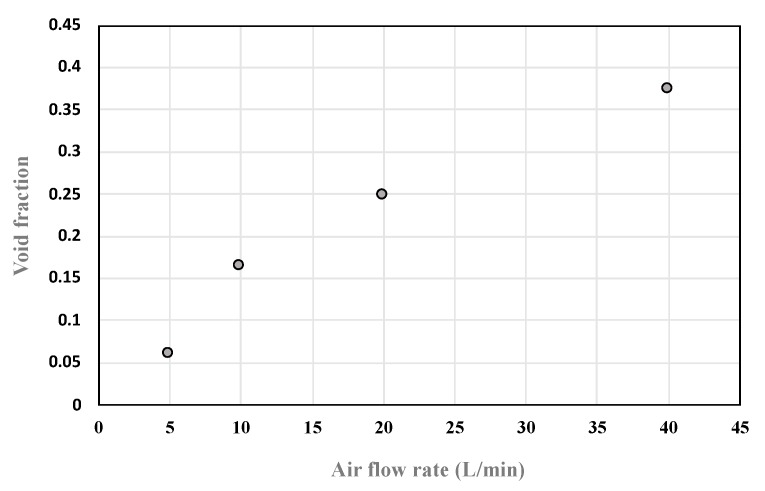
Average void fraction data points plotted versus the air flow rate.

Table 3 and Figure 27 below show the average void fraction in the tube bundle at different air flow rates using the three-sensor configuration. The results show that the average void fraction for the sensor increased as the air flow rates increased. The average void fraction for each of the three sensors was found to be quite similar (10%) at the same air flow rate. This indicates that the air bubbles are somewhat evenly distributed around the tubes, which is mainly due to the air stones fitted to the air outlet at the bottom of the model.

### 4.5. Calibration of the Sensor for Refrigerant Applications

**Figure 28:**
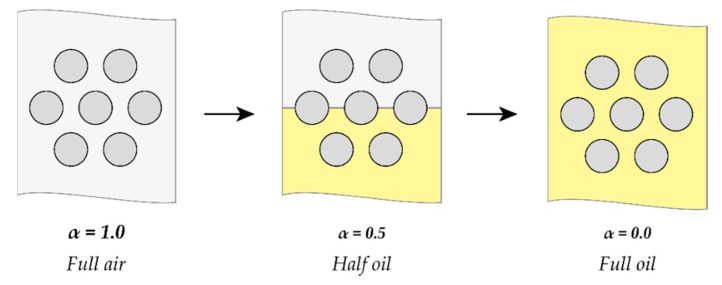
Schematic diagram of the calibration procedure.

**Figure 29:**
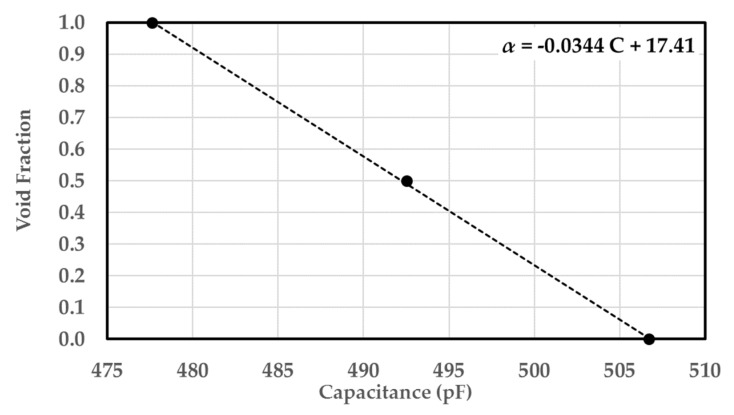
Calibration curve for air–castor oil conditions.

The sensor was first calibrated for air–castor oil in order to determine the relationship between the capacitance and the void fraction. The sampling rate was selected to be 2500 samples per second and data was collected for 10 seconds during each run. The sensor calibration was achieved by considering three points at void fractions of 0, 0.5 and 1, as shown in Figure 28. The calibration curve is shown in Figure 29. The linearity of the sensor was questioned after its length was increased. The curve showed a linear trend despite the three-inch length of the sensor. The capacitance range was found to be between 478 and 507 pF. Accordingly, the void fraction can be determined during the dynamic experiments using the calibration relation. 

**Figure 30:**
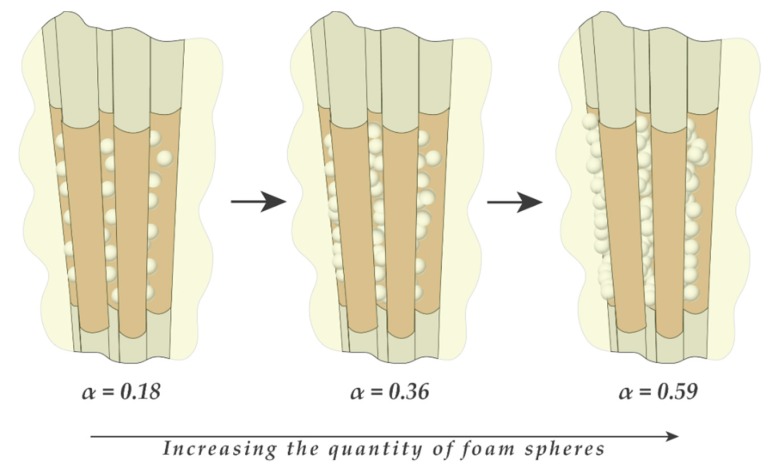
Schematic sketch of the foam spheres experiment.

**Figure 31:**
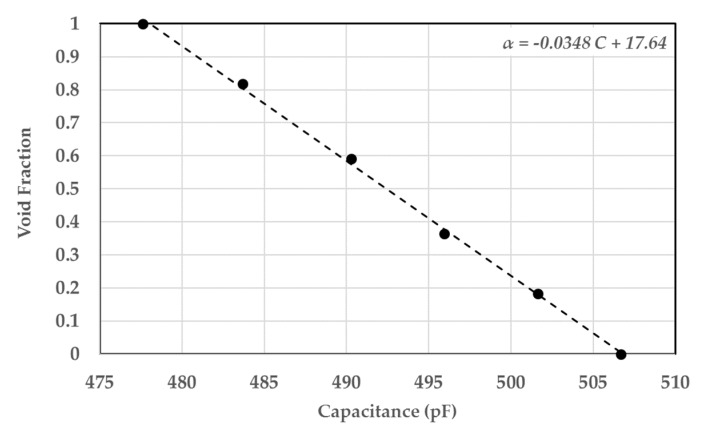
Calibration curve for air–castor oil conditions using foam spheres for the single-sensor configuration.

**Figure 32:**
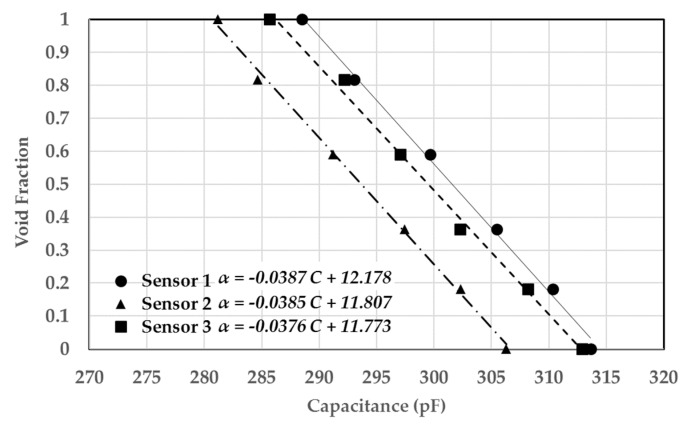
Calibration curve for air–castor oil conditions using foam spheres for the three-sensor configuration (correlation coefficient R = 0.97).

In order to simulate more realistic operating conditions for the sensor and investigate the linearity of the sensor, air bubbles were simulated using foam spheres that have similar dielectric constants. This ensures the same sensor response for gas–liquid experiments. Tiny foam spheres of diameters ranging from 2 to 4 mm were glued between the tubes, and the rest of the volume was occupied by oil, as shown in Figure 30. This allowed the experiment to determine several points on the calibration curve by increasing the number of foam spheres gradually, calculating their volume relative to the volume enclosed by the sensor, and recording the capacitance signal. The result of that is shown in Figure 31 and Figure 32 for the single-sensor configuration and the three-sensor configuration, respectively. The curves show a linear trend along six data points for both configurations with a correlation coefficient greater than 0.97. This suggests a sensor uncertainty of ±3% that was initially established by the finite element simulation. The slopes were found to be almost similar for the three sensors with very similar capacitance ranges. The data for both sensor configurations were collected during the same run to ensure consistency.

## 5. Conclusions

The present study discusses the methodology to design a capacitance sensor that can be used in measuring the void fraction of two-phase flow in tube bundles. The sensor fabrication and both static and dynamic tests of the sensor for void fraction measurements were performed using both air and water and air–castor oil to simulate refrigerant R123 cases. The experiments were conducted on a horizontal triangular tube bundle arranged in a staggered manner in order to simulate the cases of interest to the nuclear industry. The calibration of the sensor was performed in order to ensure the sensor linearity. The void fraction was determined using the average normalized capacitance values obtained from the calibration experiments. The dynamic response of the void fraction signal found to capture the actual two-phase distribution within the tube bundle confirmed by the high-speed imaging and the static calibration. The capacitance sensor was found to be as accurate as ±3% when measuring the average void fraction in tube bundles for a range between 15% and 100%. This accuracy was achieved using the calibration approach yielded results that were more accurate than the standard linear normalized capacitance approach. It should be also noted that this accuracy is lower at void fraction values below 15% due to the decrease in the electric field strength required for such low values.

## Figures and Tables

**Table 1:** Extracted fitted data from the simulation results.

Normalized Capacitance Range	Void Fraction Equation	Error (%)
0.00–0.30	α = 0.45 C*	30
0.31–0.60	α = 0.70 C* – 0.06	8.6
0.61–0.90	α = 0.95 C* – 0.20	6.3

**Table 2:** Average void fraction data for different air flow rates using the single-sensor configuration.

Air Flow Rate (L/min)	5	10	20	40
**Average Normalized Capacitance**	0.66	0.63	0.58	0.44
**Average Void Fraction**	0.071	0.077	0.211	0.389

**Table 3:** Average void fraction data for different air flow rates using the three-sensor configuration.

Air Flow Rate (L/min)	5	10	20	40
**Sensor 1**	7.60	15.75	25.47	37.14
**Sensor 2**	6.87	16.69	28.29	42.08
**Sensor 3**	8.33	18.58	26.16	37.68
